# Supplementary material for: Quantum-dot and organic hybrid tandem light-emitting diodes with multi-functionality of full-color-tunability and white-light-emission
Source: Nat Commun. 2020 Jun 4;11:2826. doi: 10.1038/s41467-020-16659-x (PMC7272409; doi:10.1038/s41467-020-16659-x)
Supplement: Supplementary file 1 — Supplementary Information [file 41467_2020_16659_MOESM1_ESM.pdf]

Supplementary Information

Quantum-dot and organic hybrid tandem light-emitting diodes with multi-functionality of full-color-tunability and white-light-emission

Zhang *et al.*

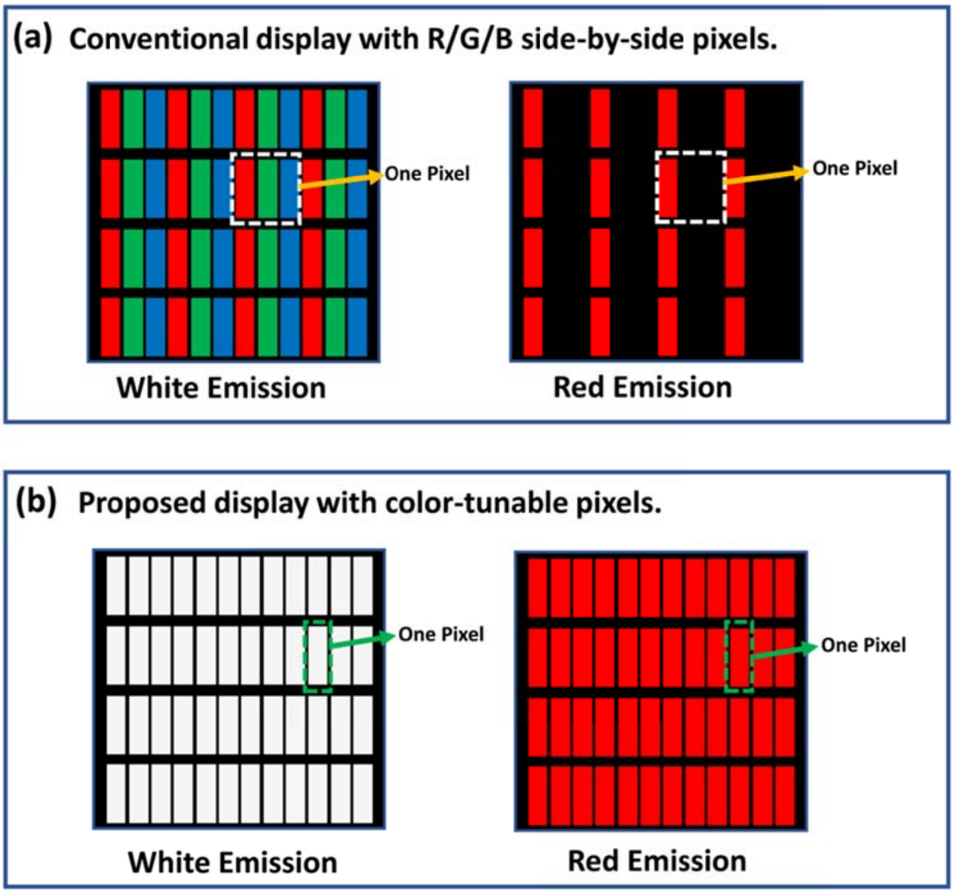

Supplementary Figure 1. Pixel arrangement of conventional display and proposed display. (a) Conventional full-color display with R/G/B side-by-side color pixels. (b) Proposed display with color-tunable pixels. A single color-tunable pixel can emit R, G and B colors, and thus can take the job of three conventional pixels. The pixel density and fill factor can be improved by threeford.

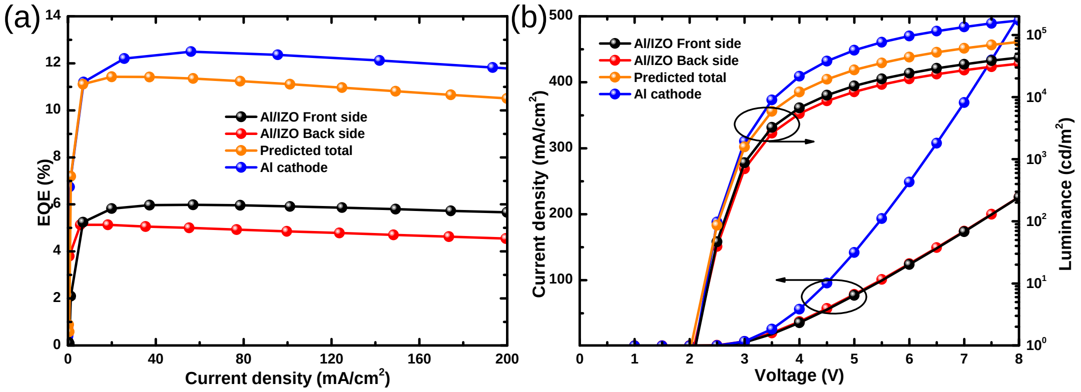

Supplementary Figure 2. EL performance of the QLED with Al or ITO top cathode. (a) The *EQE*-*J* and (b) the *J*-*V*-*L* characteristics of the R-QLEDs with Al (100 nm) and Al (2 nm)/IZO (80 nm) cathodes.

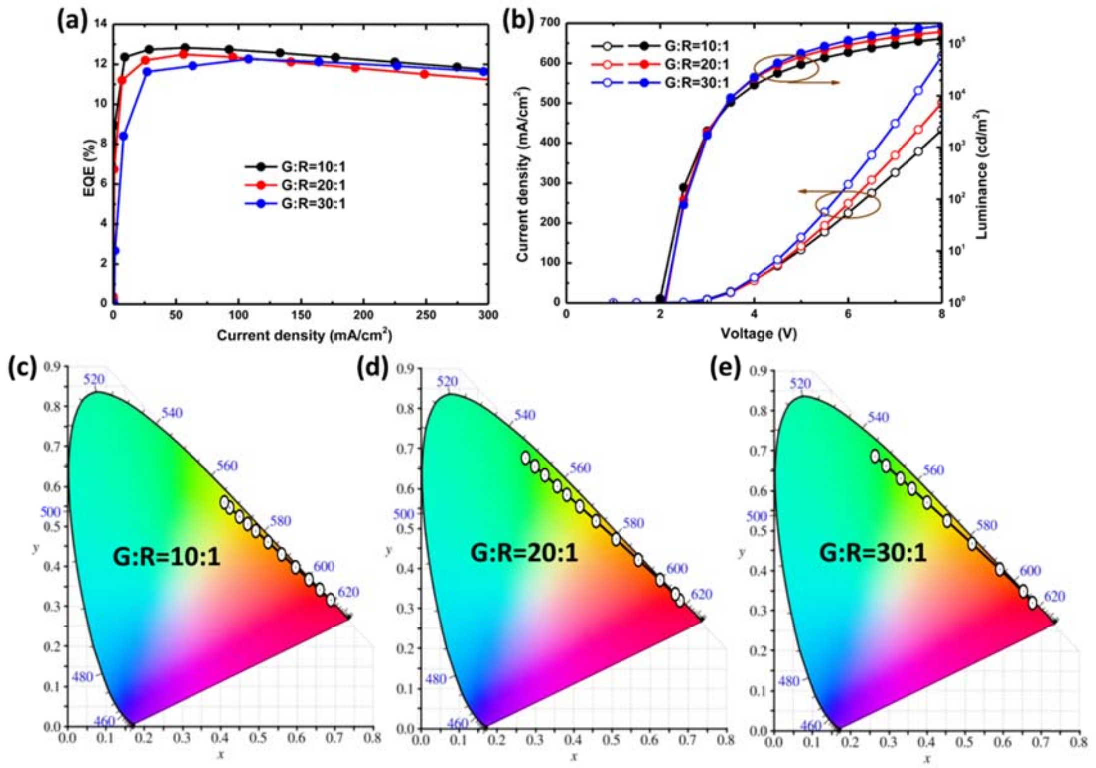

Supplementary Figure 3. Optimization of the blending ratio of G- and R-QDs. The (a) *EQE-J* and (b) *J-V-L* characteristics of the Y-QLEDs with various G-QD:R-QD ratios. The CIE color coordinates of the Y-QLEDs at 0~8 V with the G-QD:R-QD ratios of (c) 10:1, (d) 20:1 and (e) 30:1.

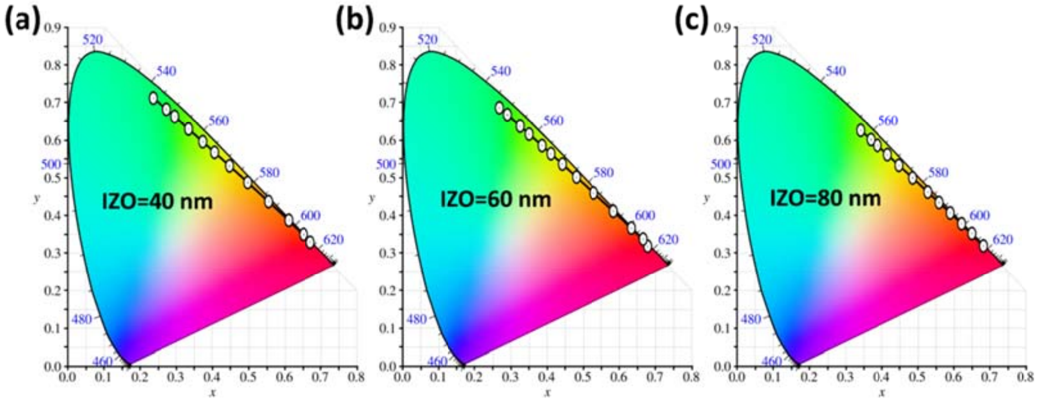

Supplementary Figure 4. Optimization of the IZO thickness. The CIE color coordinates of the Y-QLEDs at 0~8 V with a (a) 40 nm, (b) 60 nm and (c) 80 nm IZO ICE.

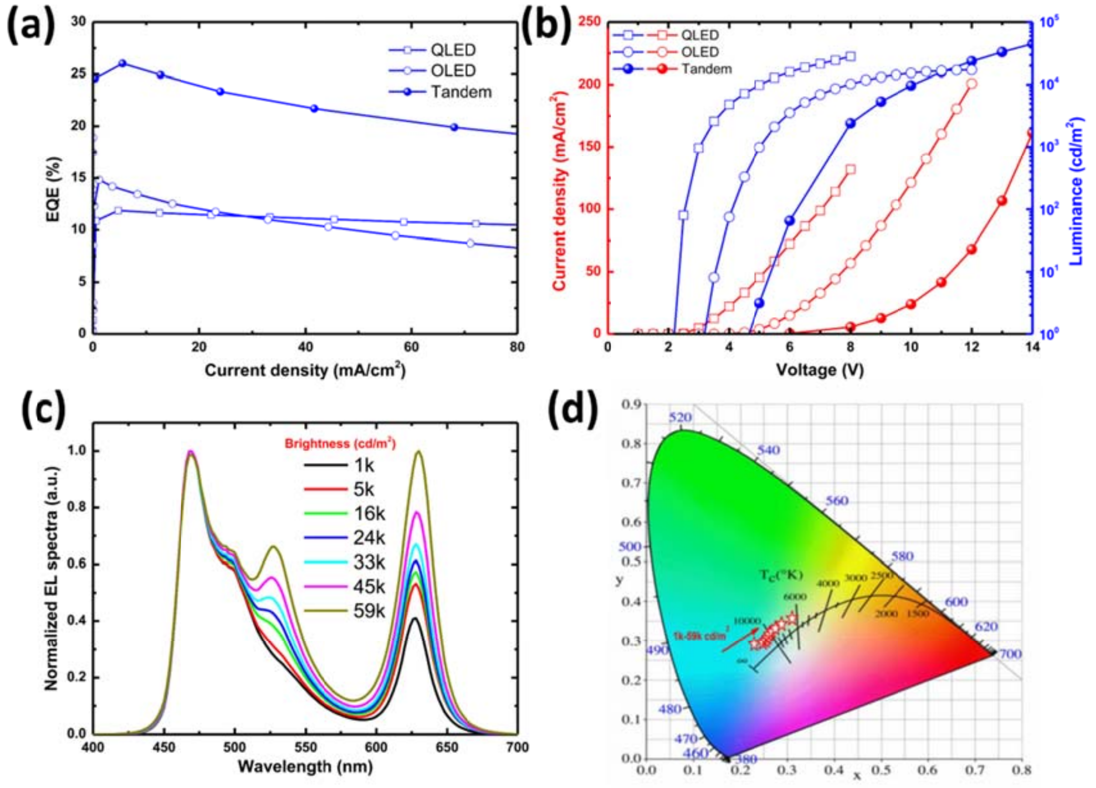

Supplementary Figure 5. EL performance of white LED with blue Ph-OLED. (a) The EQE-J and (b) J-V-L characteristics of the phosphorescent B-OLED, Y-QLED and tandem LED. (c) The normalized emission spectra and (d) CIE color coordinates of the tandem LED.

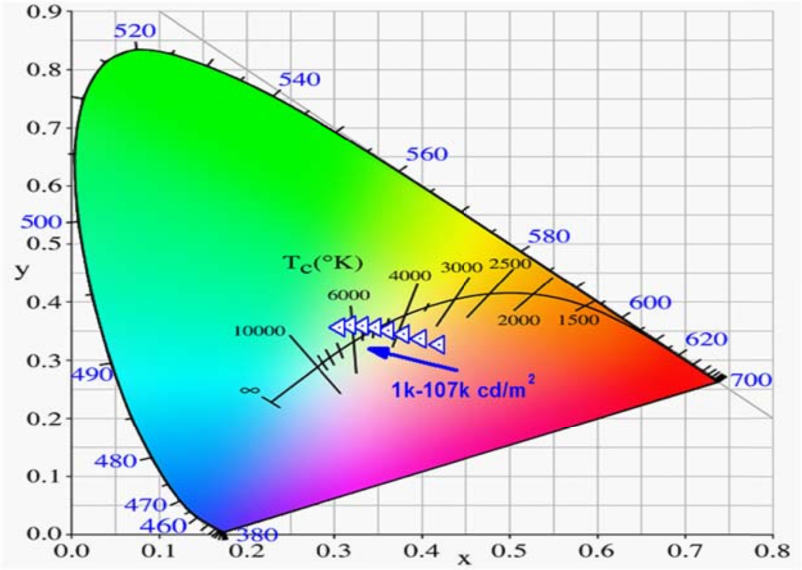

Supplementary Figure 6. The CIE coordinates of the tandem white LED when under DC driving.

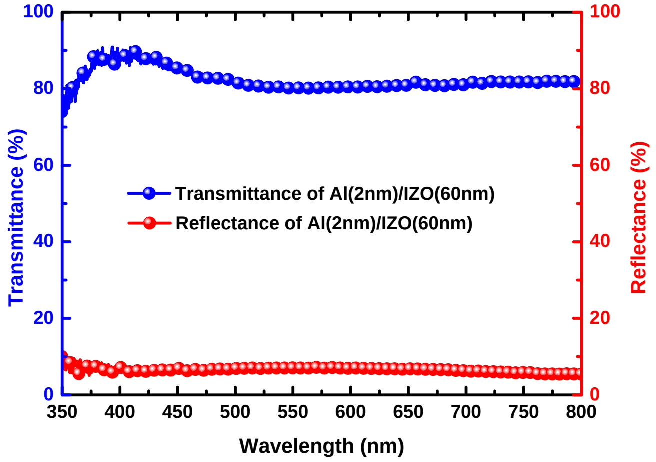

Supplementary Figure 7. The transmittance and the reflectance spectra of the Al(2 nm)/IZO(60 nm) film.

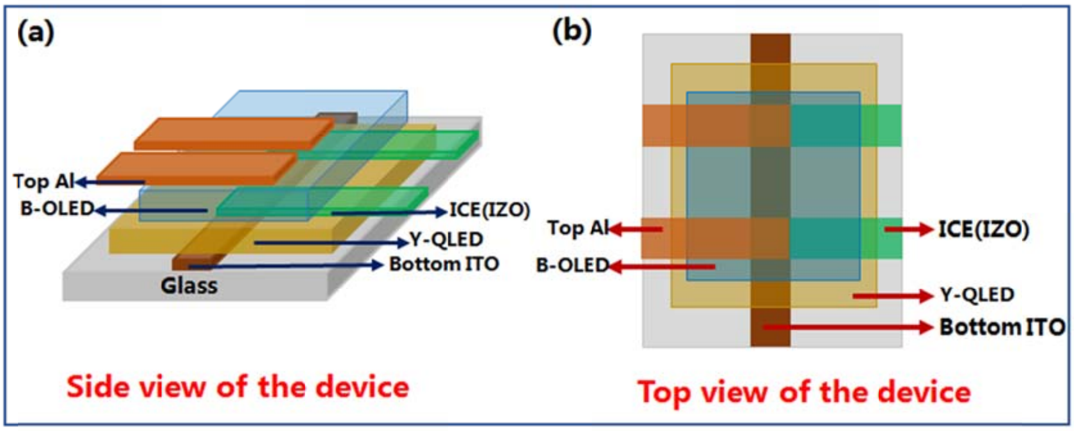

Supplementary Figure 8. The layout of the tandem LED with bottom ITO, top Al and intermediate IZO electrodes
