# Supplementary material for: Quantum-dot and organic hybrid tandem light-emitting diodes with multi-functionality of full-color-tunability and white-light-emission
Source: Nat Commun. 2020 Jun 4;11:2826. doi: 10.1038/s41467-020-16659-x (PMC7272409; doi:10.1038/s41467-020-16659-x)
Supplement: Supplementary file 3 — Description of Additional Supplementary Files [file 41467_2020_16659_MOESM3_ESM.pdf]

Description of Additional Supplementary Files

File name: Supplementary Movie 1
Description: Full-color-tunable LED. Video shows a full-color tunable LED that is configured in parallel mode and is driven by a single two-terminal alternate-current source. By changing the driving signals of the alternate-current source, the device can emit red, green and blue primary colors as well as arbitrary colors that cover a 63% National Television System Committee color triangle. The emission color can be continuously tuned from blue to red, from red to green, from green to blue and from yellow to blue.
